# Are CD4^+^CD25^-^Foxp3^+ ^cells in untreated new-onset lupus patients regulatory T cells?

**DOI:** 10.1186/ar2829

**Published:** 2009-10-12

**Authors:** Hua-xia Yang, Wen Zhang, Li-dan Zhao, Yang Li, Feng-chun Zhang, Fu-lin Tang, Wei He, Xuan Zhang

**Affiliations:** 1 Department of Rheumatology, Peking Union Medical College Hospital, Chinese Academy of Medical Sciences and Peking Union Medical College, #41 Da-Mu-Cang-Hu-Tong Street, Beijing, 100032, China; 2 Department of Immunology, School of Basic Medicine, Peking Union Medical College, and Institute of Basic Medical Sciences, Chinese Academy of Medical Sciences, #5 Dong-Dan-San-Tiao, Beijing, 100005, China

## Abstract

**Introduction:**

Our previous study has reported that, in patients with untreated new-onset lupus (UNOL), there was an abnormal increase in the number of CD4^+^CD25^-^Foxp3^+ ^T cells that correlated with disease activity and significantly decreased after treatment. However, little is known about the nature of this cell entity. The aim of this study was to explore the nature of abnormally increased CD4^+^CD25^-^Foxp3^+ ^T cells in UNOL patients.

**Methods:**

The expressions of surface (CD4, CD25, CD127, chemokine receptor 4 [CCR4], glucocorticoid-induced tumor necrosis factor receptor [GITR], and cytotoxic T lymphocyte-associated antigen 4 [CTLA-4]) and intracellular (Foxp3) molecules as well as cytokine synthesis of peripheral blood mononuclear cells from 22 UNOL patients were analyzed by flow cytometry. The proliferative and suppressive capacities of different T-cell subgroups from UNOL patients were also assessed.

**Results:**

In UNOL patients, the percentages of CD127^low/- ^in CD25^high^, CD25^low^, and CD25^- ^subpopulations of CD4^+^Foxp3^+ ^T cells were 93.79% ± 3.48%, 93.66% ± 2.31%, and 91.98% ± 2.14%, respectively (*P *> 0.05), whereas the expressions of Foxp3 showed significant differences in CD25^high ^(91.38% ± 2.57%), CD25^low ^(71.89% ± 3.31%), and CD25^- ^(9.02% ± 2.21%) subpopulations of CD4^+^CD127^low/- ^T cells (*P *< 0.01). The expressions of surface CCR4, GITR, and CTLA-4 on CD4^+^CD25^-^Foxp3^+ ^T cells were significantly less than CD4^+^CD25^+^Foxp3^+ ^T cells (*P *< 0.05). Moreover, unlike CD4^+^CD25^+^Foxp3^+ ^T cells, CD4^+^CD25^-^Foxp3^+ ^T cells also synthesized interferon-gamma, interleukin (IL)-4, IL-2, and IL-17 (*P *< 0.05), though less than CD4^+^CD25^+^Foxp3^- ^T cells. The suppressive capacity was most prominent in CD4^+^CD25^high^CD127^low/-^, followed by CD4^+^CD25^low^CD127^low/-^. CD4^+^CD25^-^CD127^- ^T cells showed the least suppressive capacity, which was similar to the effector T cells.

**Conclusions:**

CD4^+^CD25^-^Foxp3^+ ^T cells in UNOL patients are different from regulatory T cells, both phenotypically and functionally. CD127 is not an appropriate surface marker for intracellular Foxp3 in CD4^+^CD25^- ^T cells.

## Introduction

Systemic lupus erythematosus (SLE) is a systemic autoimmune disease characterized by polyclonal activation of B and T lymphocytes. It remains controversial whether the frequency and function of CD4^+^CD25^+^Foxp3^+ ^regulatory T cells (Tregs) are altered in SLE patients [1]. In our previous study, we found that, in patients with untreated new-onset lupus (UNOL), there was an abnormal increase in the number of CD4^+^CD25^-^Foxp3^+ ^T cells (instead of CD4^+^CD25^+^Foxp3^+ ^Tregs) that correlated with disease activity and significantly decreased after glucocorticoid treatment [2]. As Foxp3 is currently thought to be one of the best markers for naturally occurring Tregs (nTregs), it is intriguing to explore the nature of this abnormally increased cell entity in UNOL patients.

To answer this question requires direct functional assay and indirect phenotypic analysis. The crucial step of function assay is to find a proper surface substitute for intracellular Foxp3 in CD4^+^CD25^- ^T cells. A study has suggested that low expression of CD127 (receptor alpha chain of interleukin-7 [IL-7]) could be used as a surface marker for intracellular Foxp3 in human CD4^+^CD25^+ ^Tregs [3]. Whether this is still true in CD4^+^CD25^- ^T cells remains to be defined.

Other cell surface molecules, including glucocorticoid-induced tumor necrosis factor receptor (GITR), cytotoxic T lymphocyte-associated antigen 4 (CTLA-4), and chemokine receptor 4 (CCR4), have been investigated in Tregs. GITR has been found to be increased on CD4^+^CD25^+ ^Tregs and plays a key role in dominant immunological self-tolerance [4,5]. CTLA-4 is also predominantly expressed on CD4^+^CD25^+ ^Tregs from thymus and peripheral blood and participates in the maintenance of immunologic self-tolerance [6]. Another cell surface molecule, CCR4, is selectively expressed on Th2-type cells and Tregs [7-9]. Foxp3-transduced naïve CD4^+^CD25^- ^T cells have increased expression of CCR4 and obtain suppressive function as CD4^+^CD25^+ ^Tregs [10].

Following our report, a recent study declared that these CD4^+^CD25^-^Foxp3^+ ^T cells functionally resembled conventional Tregs by fluorescence-activated cell sorting (FACS) CD4^+^CD25^-^CD127^- ^T cells as a substitute for CD4^+^CD25^-^Foxp3^+ ^T cells from SLE patients [11]. In our current study, however, by analyzing the correlation of CD127 and Foxp3 on CD4^+^CD25^-^, CD4^+^CD25^low^, and CD4^+^CD25^high ^T cells, we found that, unlike in CD4^+^CD25^high ^T cells, CD127^low/- ^was not a perfect surface marker for intracellular Foxp3 in CD4^+^CD25^- ^T cells; therefore, CD4^+^CD25^-^CD127^low/- ^T cells could not be used as a live substitute for CD4^+^CD25^-^Foxp3^+ ^T cells to perform functional assay. We then set out to examine surface expressions of GITR, CTLA-4, and CCR4 and (importantly) cytokine synthesis function of CD4^+^CD25^-^Foxp3^+^, CD4^+^CD25^+^Foxp3^+^, and CD4^+^CD25^+^Foxp3^- ^T cells. We found that CD4^+^CD25^-^Foxp3^+ ^T cells in UNOL patients are different from Tregs, both phenotypically and functionally.

## Materials and methods

### Patients and healthy controls

Twenty-two UNOL patients of Chinese ethnicity (19 women and 3 men) were recruited in this study. All patients fulfilled the SLE classification criteria of the American College of Rheumatology. The mean age was 27.8 ± 9.1 years, and disease duration was 42 ± 28 days. Systemic lupus erythematosus disease activity index (SLEDAI) was 9.3 ± 5.2. Twenty-five gender- and age-matched healthy volunteers were involved as healthy controls. This study was approved by the ethics committee of Peking Union Medical College Hospital, and informed consent was obtained from each patient and healthy volunteer.

### Antibodies

Except as otherwise indicated, the monoclonal antibodies and reagents were obtained from eBioscience, Inc. (San Diego, CA, USA): fluorescein isothiocyanate (FITC)-conjugated anti-human CD4 (L3T4), PEcy5-conjugated anti-human CD25 (IL-2R), phycoerythrin (PE)-conjugated anti-human GITR, PE-conjugated anti-human CTLA-4, allophycocyanin-conjugated anti-human Foxp3, and PE-conjugated anti-human IL-17 and their respective isotype controls. PEcy7-conjugated CCR4, PE-conjugated anti-human interferon-gamma (IFN-γ), PE-conjugated anti-human IL-2, and PE-conjugated anti-human IL-4 and their matched isotype controls were purchased from BD Pharmingen (San Diego, CA, USA).

### Preparation of peripheral blood mononuclear cells and cell culture

Peripheral blood was collected, and peripheral blood mononuclear cells (PBMCs) were prepared by Ficoll-Hypaque density gradient centrifugation. For intracellular cytokine staining, freshly isolated PBMCs were cultured in complete RPMI 1640 media (Invitrogen Ltd., Paisley, UK) supplemented with 10% fetal bovine serum (HyClone, Logan, UT, USA), 100 U/mL penicillin, and 100 μg/L streptomycin, as well as 20 ng/mL phorbol 12-myristate 13-acetate (PMA) (Sigma-Aldrich, St. Louis, MO, USA) and 500 ng/mL ionomycin (Sigma-Aldrich), in the presence of 10 μg/mL Brefeldin A (BD Pharmingen) in a humidified CO_2_-containing atmosphere at 37°C for 6 hours.

### Flow cytometry analysis

PBMCs were washed in phosphate-buffered saline containing 2% fetal calf serum and 0.09% NaN_3_. Cells (1 × 10^6^) were incubated with FITC-CD4 (20 μL) and PEcy5-CD25 (20 μL) and with PEcy7-CCR4 (5 μL), PE-GITR (20 μL), or PE-CTLA4 (20 μL) at 4°C for 30 minutes. Subsequently, cells were perforated, and intracellular staining for Foxp3 and for PE-anti-IFN-γ (20 μL), PE-anti-IL-4 (20 μL), PE-anti-IL-2 (20 μL), or PE-anti-IL-17 (20 μL) was performed according to the instructions of the manufacturer. Stained cells were then analyzed by a FACScanto (BD Biosciences, San Jose, CA, USA).

### Functional assays

For the assessment of T-cell proliferation, FACS-sorted CD4^+^CD25^-^CD127^+^, CD4^+^CD25^high^CD127^low/-^, CD4^+^CD25^low^CD127^low/-^, and CD4^+^CD25^-^CD127^- ^from PBMCs of UNOL patients were stimulated by soluble anti-CD3 monoclonal antibody (200 ng/mL) in U-bottom 96-well plates. For the assessment of suppressive function of different T-cell subpopulations, 5 × 10^4 ^CD4^+^CD25^high^CD127^low/-^, CD4^+^CD25^low^CD127^low/-^, or CD4^+^CD25^-^CD127^- ^T cells were respectively cultured in the presence of CD4^+^CD25^-^CD127^+ ^T cells (cell ratio 1:1) and irradiated PBMCs (1 × 10^5^) in RPMI 1640 plus 10% fetal calf serum at 37°C in a humidified CO_2_-containing atmosphere for 72 hours. CCK-8 solution was added, and optical density value was measured 4 hours later.

### Statistical analysis

All statistical analyses were performed using SPSS 13.0 software (SPSS Inc., Chicago, IL, USA). Numbers of CD4^+ ^subpopulations were compared using the Student *t *test. A *P *value of less than 0.05 was considered significant.

## Results

### Correlations of CD127 and Foxp3 expressions on CD4^+^CD25^-^, CD4^+^CD25^low^, and CD4^+^CD25^high ^T cells from UNOL patients

**Figure 1:**
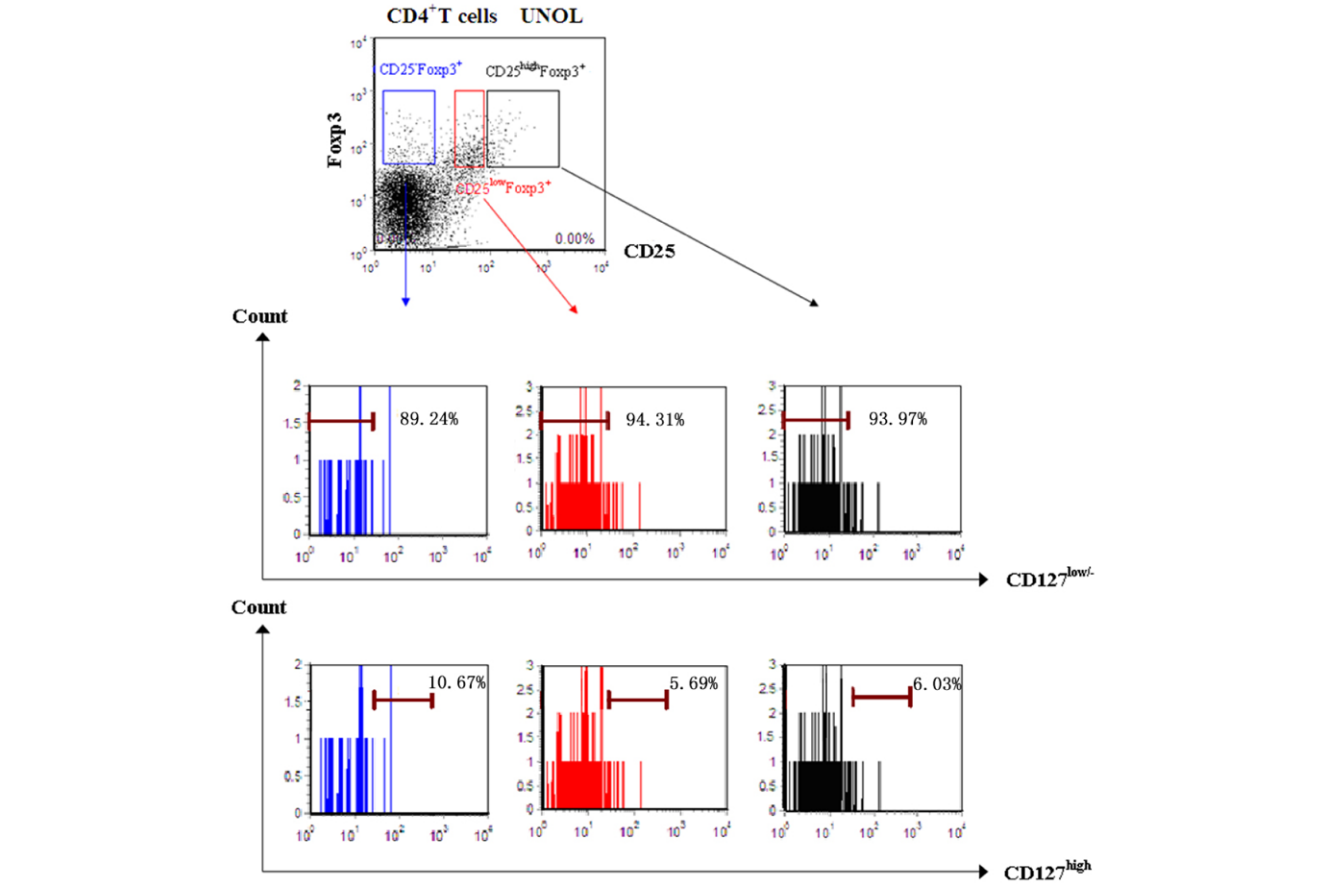
Expressions of CD127 on CD25^high^, CD25^low^, and CD25^- ^subpopulations of CD4^+ ^Foxp3^+ ^T cells from patients with untreated new-onset lupus (UNOL).

**Figure 2:**
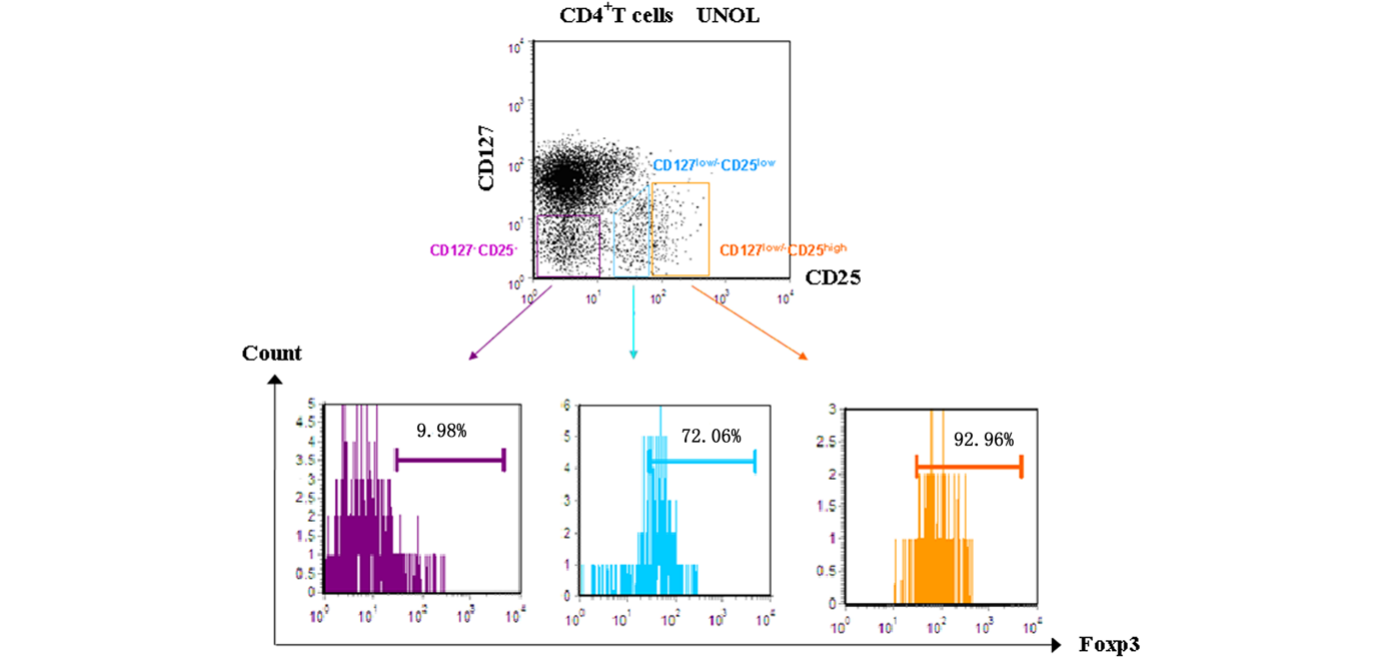
Expressions of Foxp3 in CD25^high^, CD25^low^, and CD25^- ^subpopulations of CD4^+ ^CD127^low/- ^T cells from patients with untreated new-onset lupus (UNOL).

CD4^+ ^T cells were divided into three subgroups by CD25 expression: CD4^+^CD25^high^, CD4^+^CD25^low^, and CD4^+^CD25^- ^T cells. We gated CD127^low/- ^expression on Foxp3^+ ^T cells and backgated Foxp3 expression on CD127^low/- ^T cells, respectively. We found that all CD4^+^Foxp3^+ ^T cells had a low expression level of CD127, regardless of CD25 expression. Percentages of CD127^low/- ^in CD25^high^, CD25^low^, and CD25^- ^subpopulations of CD4^+^Foxp3^+ ^T cells were 93.79% ± 3.48%, 93.66% ± 2.31%, and 91.98% ± 2.14%, respectively (*P *> 0.05) (Figure 1). On the other hand, the expressions of Foxp3 on CD4^+^CD127^low/- ^T cells showed significant differences in CD25^high ^(91.38% ± 2.57%), CD25^low ^(71.89% ± 3.31%), and CD25^- ^(9.02% ± 2.21%) subpopulations (*P *< 0.01) (Figure 2). Foxp3 expressions in CD4^+^CD127^low/- ^T cells were high in CD25^high ^but low in CD25^- ^subpopulations. This result suggested that, unlike in CD4^+^CD25^high ^T cells, CD127^low/- ^was not a perfect candidate surface marker for intracellular Foxp3 in CD4^+^CD25^- ^T cells and that CD4^+^CD25^-^CD127^low/- ^T cells could not be used as a live substitute for CD4^+^CD25^-^Foxp3^+ ^T cells to perform functional assay.

Expressions of GITR, CTLA-4, CCR4 and effector T cell-related cytokines on CD4^+ ^subpopulations from UNOL patients

### Expressions of GITR, CTLA-4, CCR4, and effector T cell-related cytokines (IFN-γ, IL-4, IL-2, and IL-17) on CD4^+^CD25^-^Foxp3^+^, CD4^+^CD25^+^Foxp3^-^, CD4^+^CD25^+^Foxp3^+^, and CD4^+^CD25^-^Foxp3^- ^T cells from UNOL patients and healthy controls

**Figure 3:**
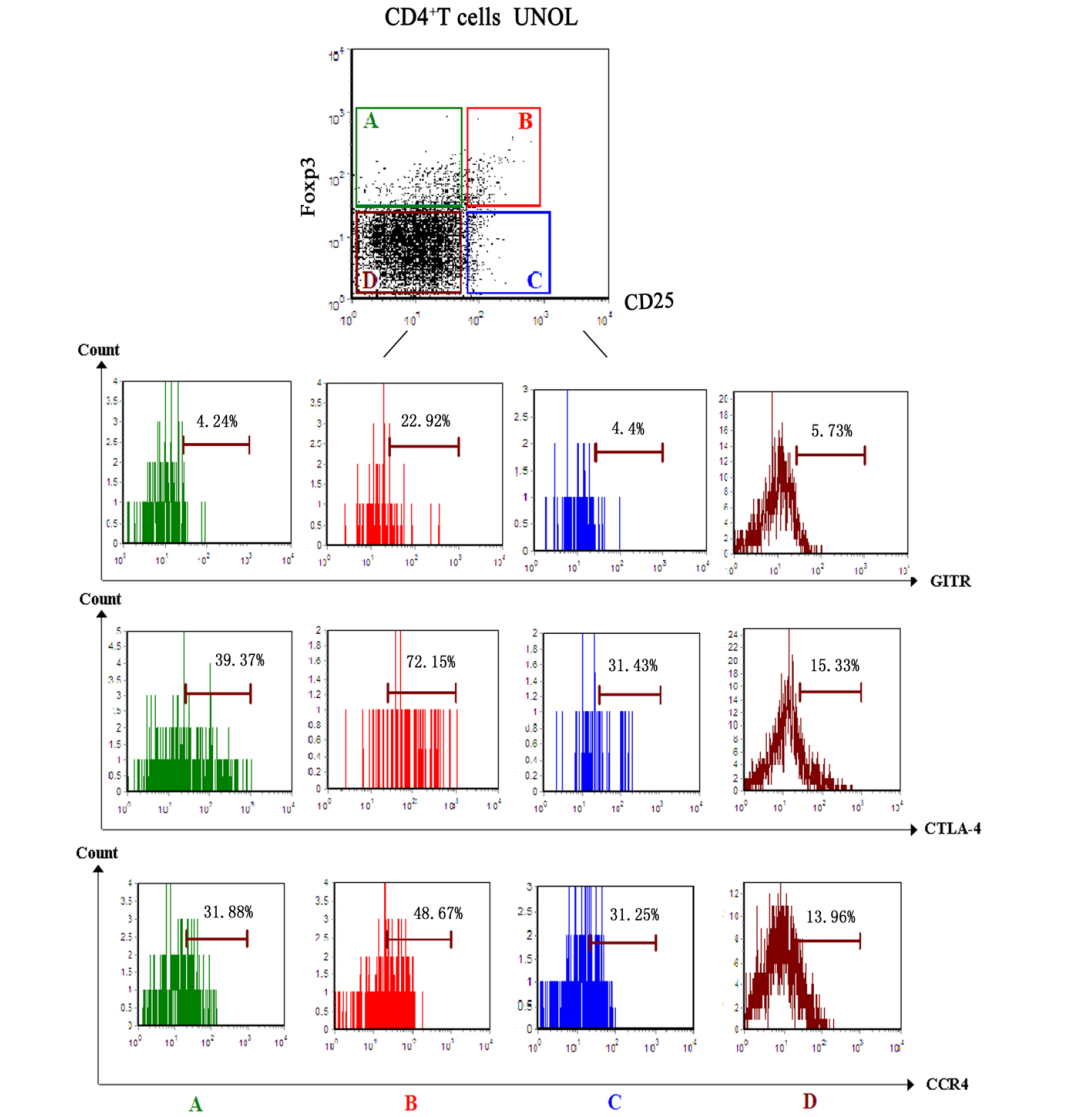
Expressions of glucocorticoid-induced tumor necrosis factor receptor (GITR), cytotoxic T lymphocyte-associated antigen 4 (CTLA-4), and chemokine receptor 4 (CCR4) on CD4^+ ^subpopulations from patients with untreated new-onset lupus (UNOL). **(A) **CD4^+ ^CD25^- ^Foxp3^+^. **(B) **CD4^+ ^CD25^+ ^Foxp3^+^. **(C) **CD4^+ ^CD25^+ ^Foxp3^-^. **(D) **CD4^+ ^CD25^- ^Foxp3^-^.

**Figure 4:**
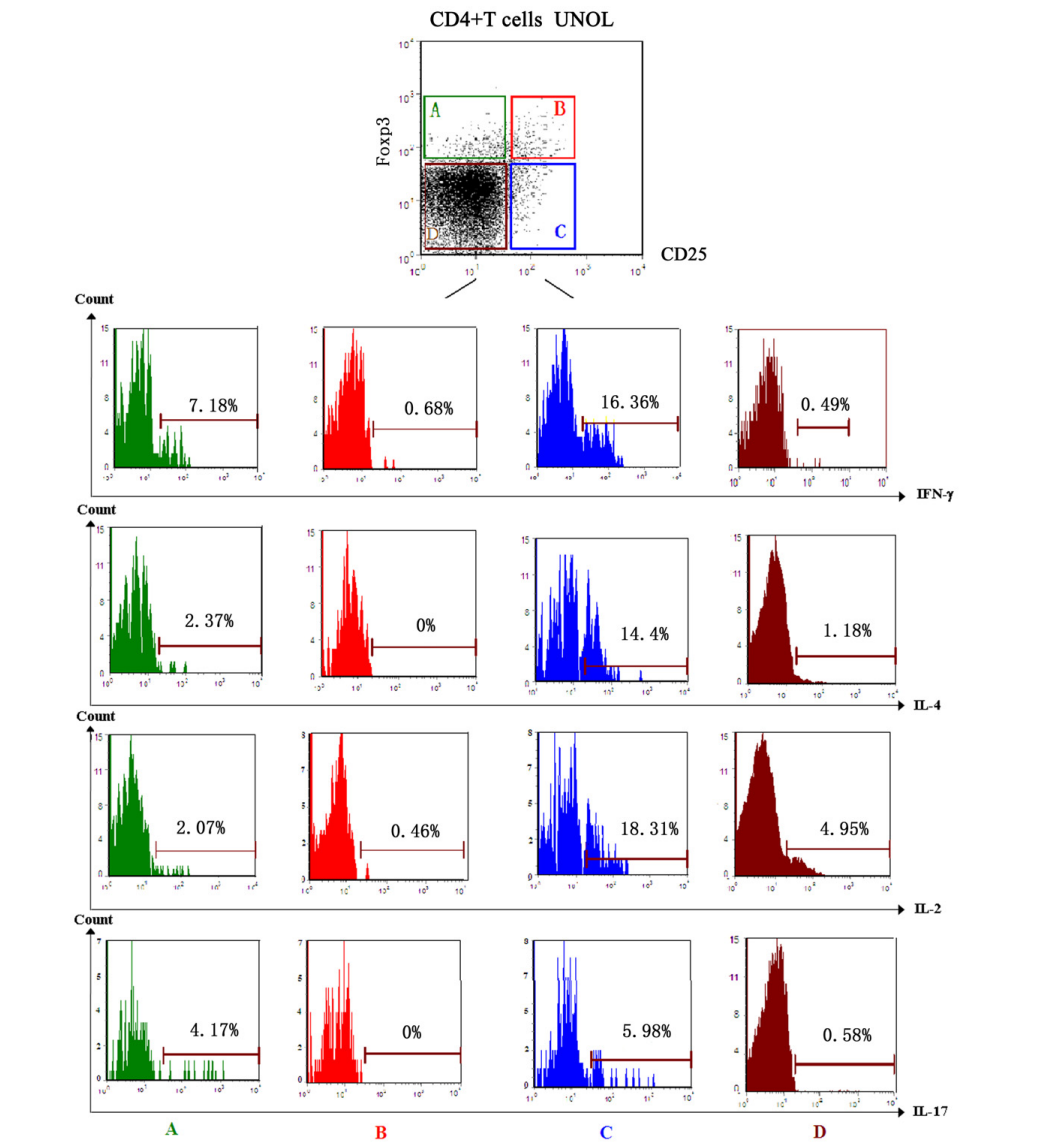
Expressions of interferon-gamma (IFN-γ), interleukin (IL)-4, IL-2, and IL-17 on CD4^+ ^subpopulations from patients with untreated new-onset lupus (UNOL). **(A) **CD4^+ ^CD25^- ^Foxp3^+^. **(B) **CD4^+ ^CD25^+ ^Foxp3^+^. **(C) **CD4^+ ^CD25^+ ^Foxp3^-^. **(D) **CD4^+ ^CD25^- ^Foxp3^-^.

As shown in Table 1 and Figure 3, in UNOL patients, there was no significant difference between CD4^+^CD25^-^Foxp3^+ ^and CD4^+^CD25^+^Foxp3^- ^T cells in the expressions of GITR, CTLA-4, and CCR4 (*P *> 0.05), whereas they were both less than CD4^+^CD25^+^Foxp3^+ ^T cells (*P *< 0.01). Moreover, the expressions of effector T cell (Teff)-related cytokines, including IFN-γ, IL-4, IL-2, and IL-17, were analyzed to examine cytokine synthesis capacity of CD4^+^CD25^-^Foxp3^+ ^T cells. As shown in Table 2 and Figure 4, in UNOL patients, unlike Tregs (CD4^+^CD25^+^Foxp3^+^), CD4^+^CD25^-^Foxp3^+ ^T cells also synthesized IFN-γ, IL-4, IL-2, and IL-17 (*P *< 0.05), though less than Teffs (CD4^+^CD25^+^Foxp3^-^).

**Table 1:** Expressions of GITR, CTLA-4, and CCR4 on CD4^+ ^subpopulations from untreated new-onset lupus patients and healthy controls

Subgroups	GITR, %		CTLA-4, %		CCR4, %	
	UNOL	HC	UNOL	HC	UNOL	HC
CD4^+ ^CD25^- ^Foxp3^+^	4.41 ± 0.67	5.13 ± 1.23	39.78 ± 1.67	53.12 ± 4.29	35.76 ± 2.53	34.33 ± 2.90
CD4^+ ^CD25^+ ^Foxp3^+^	22.49 ± 1.75	29.88 ± 3.24	73.89 ± 2.76	81.66 ± 4.85	49.44 ± 2.75	56.91 ± 3.17
CD4^+ ^CD25^+ ^Foxp3^-^	6.52 ± 0.89	4.89 ± 1.32	33.57 ± 2.98	40.59 ± 5.55	31.99 ± 3.76	32.23 ± 5.54
CD4^+ ^CD25^- ^Foxp3^-^	5.35 ± 0.88	11.77 ± 2.75	15.05 ± 2.24	16.06 ± 4.25	13.58 ± 2.57	10.11 ± 3.63

Values are presented as mean ± standard deviation. CCR4, chemokine receptor 4; CTLA-4: Cytotoxic T lymphocyte-associated antigen 4; GITR: glucocorticoid-induced tumor necrosis factor receptor; HC: healthy controls; UNOL: patients with untreated new-onset lupus.

**Table 2:** Expressions of IFN-γ, IL-4, IL-2, and IL-17 on CD4^+ ^subpopulations from untreated new-onset lupus patients and healthy controls

	IFN-γ, %		IL-4, %		IL-2, %		IL-17, %	
	
	UNOL	HC	UNOL	HC	UNOL	HC	UNOL	HC
CD4^+ ^CD25^- ^Foxp3^+^	7.56 ± 1.23	5.79 ± 1.05	2.97 ± 0.83	2.02 ± 0.83	3.59 ± 1.95	5.09 ± 1.95	4.61 ± 1.54	1.54 ± 1.02

CD4^+ ^CD25^+ ^Foxp3^+^	0.72 ± 0.34	1.22 ± 0.58	0.39 ± 0.37	0.88 ± 0.37	0.73 ± 0.49	0.22 ± 0.49	0.38 ± 0.32	0.08 ± 0.06

CD4^+ ^CD25^+ ^Foxp3^-^	16.43 ± 3.51	16.81 ± 3.97	13.15 ± 2.99	12.94 ± 2.99	20.41 ± 4.91	19.91 ± 4.91	5.58 ± 1.51	2.37 ± 1.51

CD4^+ ^CD25^- ^Foxp3^-^	3.54 ± 1.05	5.92 ± 1.57	0.94 ± 0.56	0.66 ± 0.56	2.92 ± 1.42	8.22 ± 1.42	0.49 ± 0.35	0.67 ± 0.42

Values are presented as mean ± standard deviation. HC: healthy controls; IFN-γ: interferon-gamma; IL: interleukin; UNOL: patients with untreated new-onset lupus.

### Functional assays of T-cell subgroups from UNOL patients

**Figure 5:**
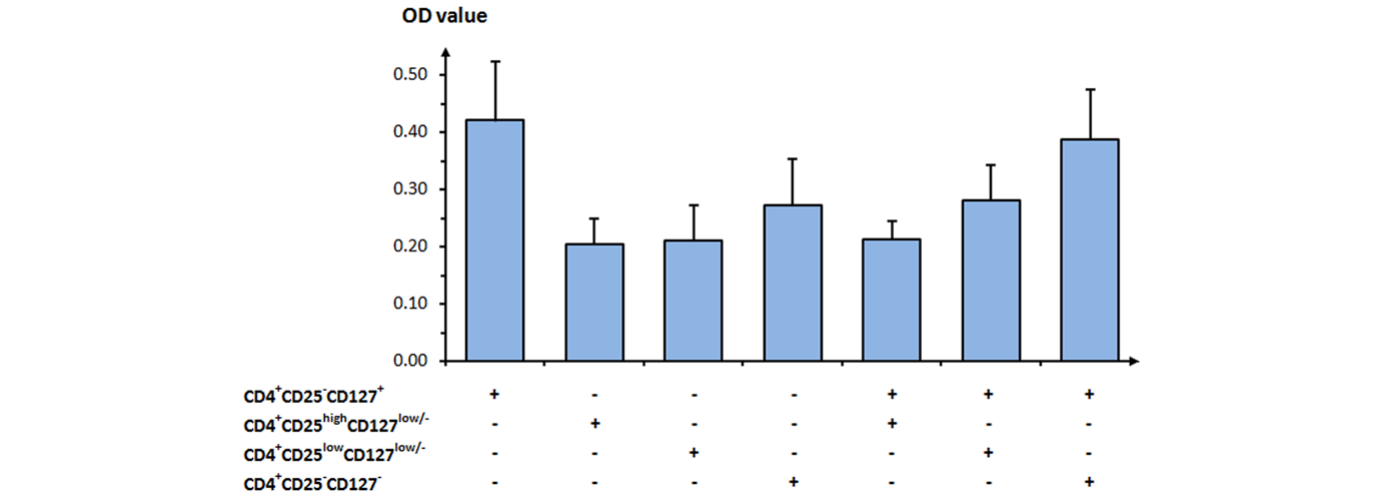
Assessment of proliferative and suppressive capacities of CD4^+ ^CD25^- ^CD127^+^, CD4^+ ^CD25^high^CD127^low/-^, CD4^+ ^CD25^low^CD127^low/-^, and CD4^+ ^CD25^- ^CD127^- ^T cells from patients with untreated new-onset lupus. Values are presented as mean ± standard error of the mean (n = 8). OD, optical density.

CD4^+^CD25^-^CD127^+ ^(Teffs), CD4^+^CD25^high^CD127^low/- ^(Tregs), CD4^+^CD25^low^CD127^low/-^, and CD4^+^CD25^-^CD127^- ^T cells from UNOL patients were sorted respectively. First, all four subgroups were stimulated with anti-CD3 and assessed for their proliferative ability. CD4^+^CD25^high^CD127^low/- ^Tregs (0.205 ± 0.043) were found to be anergic compared with CD4^+^CD25^-^CD127^+ ^Teffs (0.421 ± 0.102). Similarly, CD4^+^CD25^low^CD127^low/- ^(0.210 ± 0.062) and CD4^+^CD25^-^CD127^- ^(0.272 ± 0.081) T cells showed a reduced proliferative response (Figure 5).

Then, CD4^+^CD25^high^CD127^low/-^, CD4^+^CD25^low^CD127^low/-^, and CD4^+^CD25^-^CD127^- ^T cells were respectively cocultured with CD4^+^CD25^-^CD127^+ ^Teffs. The suppressive capacity as shown by optical density was most prominent in CD4^+^CD25^high^CD127^low/- ^(0.213 ± 0.032), followed by CD4^+^CD25^low^CD127^low/- ^(0.281 ± 0.061) and CD4^+^CD25^-^CD127^- ^(0.387 ± 0.087). CD4^+^CD25^-^CD127^- ^T cells showed the least suppressive capacity, which was similar to the Teffs, in line with its lesser expression of Foxp3 (9.02% ± 2.21%) (Figure 5).

## Discussion

Foxp3 is currently thought to be one of the best markers for nTregs. It plays a pivotal role in the development and maturation of Tregs. Foxp3-deficient mice develop systemic autoimmune disease, and evidence from adoptive transfer experiments suggests that this is the direct result of nTreg defect. Moreover, overexpression of Foxp3 in murine CD4^+ ^T cells is sufficient to generate Tregs *in vitro*. In humans, Foxp3 deficiency also leads to a systemic autoimmune disease known as IPEX (immune dysregulation, polyendocrinopathy, enteropathy X-linked syndrome). It has been shown, however, that the expression of Foxp3 is necessary, but not sufficient, to confer regulatory function of Tregs. Foxp3 is also expressed on some activated CD4^+ ^T cells [12]. Bonelli and colleagues [13] reported that Foxp3 expression on CD4^+ ^T cells significantly correlated with CD69 expression and that Foxp3 might be associated with T-cell activation.

In our previous study, we found that a significant increase of CD4^+^CD25^-^Foxp3^+ ^T cells in UNOL patients correlated with disease activity and that the cell number significantly decreased after glucocorticoid treatment [2]. Whether these cells are Tregs or activated Teffs remains to be determined. Functional assay would be a direct way to identify the nature of CD4^+^CD25^-^Foxp3^+ ^T cells if only we could find a proper surface substitute for intracellular Foxp3 in CD4^+^CD25^- ^T cells. A study showed that low expression of CD127 could be used as a surface marker for intracellular Foxp3 in human CD4^+^CD25^+ ^Tregs [3]. CD127 is expressed not only on lymphocytes, but also on monocytes and dendritic cells. Its ligand, IL-7, is a pivotal cytokine involved in the development and survival of T and B lymphocytes [14]. In addition, thymic stromal lymphopoietin (TSLP) signals through CD127 in a heterodimeric complex with TSLP receptor [15]. TSLP-activated dendritic cells might participate in the homeostatic maintenance of CD4^+ ^and development of Tregs in thymus [16].

In this study, we gated and backgated expressions of CD127 and Foxp3 in CD4^+^CD25^- ^T cells. We confirmed that CD4^+^CD25^high^CD127^low/- ^could be used as a substitute for isolating CD4^+^CD25^high^Foxp3^+ ^Tregs, whereas the expression of Foxp3 on CD4^+^CD127^low/- ^T cells showed significant differences in CD25^high ^(91.38% ± 2.57%), CD25^low ^(71.89% ± 3.31%), and CD25^- ^(9.02% ± 2.21%) subpopulations. Foxp3 expression on CD4^+^CD127^low/- ^T cells was high in both CD25^high ^and CD25^low ^subpopulations but low in CD25^- ^subpopulations. This result suggested that, unlike in CD4^+^CD25^high ^T cells, CD127 was not a perfect surface marker for intracellular Foxp3 in CD4^+^CD25^- ^T cells. It is also important to note that, although the CD25^low ^population lies adjacent to CD25^- ^on a FACS plot (as shown in Figure 2), they belong to two different cell entities as their Foxp3 expressions as well as their suppressive capacity and response to *in vitro *stimulation were different. If the sorted CD25^- ^subgroup was 'contaminated' with CD25^low^, it would bias function analysis of CD4^+^CD25^-^Foxp3^+ ^T cells from Teffs to Tregs [11].

Another possible explanation of the differences between the study of Bonelli and colleagues [13] and ours is that there may be a difference between untreated, newly diagnosed patients and those more chronically ill who were drawn from an outpatient population. It is possible that, as a consequence of illness, true CD25^+ ^Tregs have become CD25^-^, whereas this has not occurred yet in patients with new-onset disease.

In our study, we found that the expressions of GITA, CTLA-4, and CCR4 on CD4^+^CD25^-^Foxp3^+ ^T cells resembled CD4^+^CD25^+^Foxp3^- ^Teffs and were significantly less than CD4^+^CD25^+^Foxp3^+ ^Tregs. Moreover, unlike CD4^+^CD25^+^Foxp3^+ ^Tregs, CD4^+^CD25^-^Foxp3^+ ^T cells also synthesized IFN-γ, IL-4, IL-2, and IL-17, though less than CD4^+^CD25^+^Foxp3^-^Teffs, suggesting that the abnormally increased CD4^+^CD25^-^Foxp3^+ ^T cells in UNOL patients were not simple and pure Tregs.

## Conclusions

CD4^+^CD25^-^Foxp3^+ ^T cells in UNOL patients are different from Tregs, both phenotypically and functionally. CD127 is not an appropriate surface marker for intracellular Foxp3 in CD4^+^CD25^- ^T cells.

## Abbreviations

CCR4: chemokine receptor 4; CTLA-4: cytotoxic T lymphocyte-associated antigen 4; FACS: fluorescence-activated cell sorting; FITC: fluorescein isothiocyanate; GITR: glucocorticoid-induced tumor necrosis factor receptor; IFN-γ: interferon-gamma; IL: interleukin; nTreg: naturally occurring regulatory T cell; PBMC: peripheral blood mononuclear cell; PE: phycoerythrin; SLE: systemic lupus erythematosus; Teff: effector T cell; Treg: regulatory T cell; TSLP: thymic stromal lymphopoietin; UNOL: untreated new-onset lupus.

## Competing interests

The authors declare that they have no competing interests.

## Authors' contributions

HY and WZ developed the study, analyzed the data, and drafted the manuscript. LZ and YL participated in the data collection, performed the data analysis, and helped in the drafting of the manuscript. XZ and FZ participated in the development of the study, data analysis, and the drafting of the manuscript. FT and WH conceived the study and drafted the manuscript. All authors have read and approved the manuscript.
